# Editorial: Intestinal Microbiota in the Pathogenesis and Management of Necrotizing Enterocolitis in Preterm Infants

**DOI:** 10.3389/fped.2022.837925

**Published:** 2022-02-17

**Authors:** Chunbao Guo, Yuan Shi

**Affiliations:** ^1^Department of Pediatric General Surgery, Children's Hospital of Chongqing Medical University, Chongqing, China; ^2^Ministry of Education Key Laboratory of Child Development and Disorders, National Clinical Research Center for Child Health and Disorders, Children's Hospital of Chongqing Medical University, Chongqing, China; ^3^China International Science and Technology Cooperation Base of Child Development and Critical Disorders, Chongqing Key Laboratory of Pediatrics, Children's Hospital of Chongqing Medical University, Chongqing, China; ^4^Department of Neonatology, Chongqing Medical University, Chongqing, China

**Keywords:** necrotizing enterocolitis, intestinal microbiome, bioinformatic processing analysis, antibiotic management, probiotic prophylaxis, intrauterine hypoxia, gut microbiome dysbiosis

Necrotizing enterocolitis (NEC) is the most fatal disease involving preterm infants, with substantial mortality (20–35%) as well as long-term morbidity in terms of gastrointestinal and neurodevelopmental symptoms. The incidence of NEC might be up to 10% for infants weighing <1,500 g (1). Approximately 10–100 trillion symbiotic bacteria constitute the human microbiome, most of which reside within the gastrointestinal tract. The neonatal period is essential for human microbiome development, which might contribute to many disease processes, including NEC (2).

Multiple studies have demonstrated a decrease in bacterial diversity and a Gammaproteobacteria bloom in the intestinal microbiota several weeks prior to the onset of NEC in preterm infants compared with term infants. Accordingly, the pathogenesis of NEC has been associated with microbial dysbiosis, which is characterized by an altered microbial community and microbial invasion of the gut mucosa with excessive inflammation, particularly in preterm infants (3). Environmental pressures should contribute to microbial dysbiosis in preterm infants, such as instrumentation, antibiotic stress, and neonatal intensive care unit (NICU) environments (4).

For necrotizing enterocolitis, the main therapeutic focus should be restoring the microbial balance to construct a healthy microbial community with optimization of beneficial organisms and pathogens (5). However, inconsistent results are usually reported, and they under power these concepts. For neonatal intestinal microbiome evaluation, technological advances have provided greater resolution and availability (6). With the current topic, we intend to provide more sound evidence for all areas of research involving the intestinal microbiome in the pathogenesis, diagnosis and management of NEC. Here, we published a special collection of 10 articles contributing to this concept.

Valuable insights into the intestinal microbiome for many disease processes, including NEC, are increasing (7). A clearer understanding of the healthy neonatal microbiome composition and the altered microbial community in preterm infants should be very important for its pathogenesis (8). First and foremost, a consistent pattern over time in microbiome composition with many different species has been presented in premature NEC infants. Here, in the current collection, several studies assessed the intestinal microbiome under various conditions utilizing 16S ribosomal ribonucleic acid (rRNA) sequence techniques among preterm infants.

Fu et al. emphasized the importance of the association between intestinal microbiota and NEC following bioinformatic processing analysis. They note that the microbial species varies following NEC occurrence and that multiple communities are critical for NEC development. NEC infants presented with a higher abundance of waterborne bacteria, which live in the environment. Li et al. found a significant difference in diversity of the intestinal flora after feeding intolerance recovery from 11 pairs of premature twins/triplets following the 16S rRNA gene sequencing. We find their approach applaudable and absolutely agree with the authors' point. Therefore, attention should be given to contamination during disease care and management within the NICU. They further proposed the prospect that in-depth investigation of microbial colonization could detect the mechanism of NEC.

The intestinal microbiome should be influenced by various factors in premature infants who experience NEC due to their disease severity. Recently, clinical research has confirmed that many prenatal and postnatal factors might adversely affect the gut microbial balance, including formula feeding, antibiotic management and mode of delivery (9). However, no randomized clinical trials (RCTs) are currently available to investigate the influence of various environmental and care conditions on the intestinal microbiome in premature infants. In this collection, three articles involve the association of gut microbial balance with family integrated care, intrauterine hypoxia and antibiotic regimens. Yang et al. present their prospective pilot study to evaluate the effect of family integrated care on intestinal flora diversity and found that short-term family integrated care did not significantly influence the establishment of gut microbial balance in premature NEC infants due to a small sample size. They recommend an adequately powered clinical trial to make a conclusion.

Intrauterine hypoxia is the most important cause of hypoxic-ischemic encephalopathy in infants, fetal intrauterine distress, growth restriction, and even fetal or neonatal death (10). Furthermore, intrauterine hypoxia could influence microbiota diversity formation, especially anaerobic bacterial colonization within the gastrointestinal tract. Sun et al. performed an experimental NEC study involving hypoxic conditions and indicated that intrauterine hypoxia could greatly promote the occurrence of NEC through disruption of the gut microbiota balance. Therefore, for infants born with intrauterine hypoxia, more caution should be taken for the monitoring of abdominal conditions, and careful feeding management should be required.

Studies have suggested that long-term antibiotic administration in infants could have a great impact on the composition of the normal intestinal microbial community, which could further interfere with metabolic and immune balance. The quick cessation of antibiotic management could restore microbiota diversity (11). In this study, Chang et al. further demonstrated that in VLBW preterm infants, antibiotic management had an essential influence on the structure of gut microbiota. Overabundance of Enterococcus could be present following usage of ampicillin and cefotaxime for prolonged periods. Unfortunately, despite previous exposure to antibiotics, various bacterial genera could still colonize the gastrointestinal tract, which might be harmful for preterm infants.

Han et al. found that infectious disease conditions were also related to the gut microbiota of neonates, such as pneumonia or meningitis. The initial gut microbiome is associated with its state following 1 week of antibiotic management. Under these conditions, antibiotic administration within 7 days had little effect on the composition of gut microbiota and community richness.

To construct the patient's own bacterial ecosystem with beneficial commensal bacteria, the use of prebiotic and/or probiotic administration has blossomed. Live microbiota (probiotics) could prevent gut microbiome dysbiosis through inhibition of pathogenic bacteria and promotion of beneficial bacterial colonization, and its use has been proven safe and effective in NEC management. To date, probiotic prophylaxis administration to preterm infants has been widely adopted in the NICU (12). However, probiotic utilization in preterm infants in China remains relatively limited. Furthermore, the administration protocols, combination strains and synergistic interactions might all lead to challenges in optimal probiotic selection for clinical usage. Meanwhile, food substrates within human milk, such as lactalbumin, lactoferrin inulin, or oligosaccharides, should be supplied to facilitate commensal flora reproduction and its activity.

The present research conducted by Juber et al. demonstrated that routine multispecies probiotic supplementation containing Bifidobacterium and Lactobacillus had a limited effect on the incidences of NEC and its mortality among infants with very low birth weight, which contrasts with multiple other studies, even with the exact same probiotic strain. Priyadarshi et al. found similar clinical outcomes over a 4-year period in preterm infants between the single- or two-strain probiotic prophylaxis strategy, including NEC incidence, feeding outcomes, and comorbidities. The different conclusions might have resulted from the limited sample size and relatively low baseline NEC incidences, which could not be defined with the threshold of probiotic efficacy. Further investigations with large-scale randomized controlled multicenter trials are needed to definitively determine the strain-specific effects in reducing NEC and mortality in preterm infants. A microbiome study of stool specimens should also be performed to determine the beneficial effects of intestinal colonization.

Understanding the exact mechanism of pathogenic bacterial strains in relation to NEC is critically important to produce and sustain a stable intestinal microbiota ecosystem (8). Cassir et al. report here a case of *Clostridium neonatale* bacteremia in a preterm neonate with NEC, suggesting its potential role in NEC pathogenesis. They emphasize that the sporulating form of *C. neonatale* could confer resistance to usual hospital environmental cleaning measures. The causal relationship between the isolation of *C. neonatale* and the occurrence of NEC should be further investigated, and it should be promising in diagnostic and therapeutic management.

Finally, the early diagnosis of NEC is challenging due to the non-specific clinical characteristics of more immature individuals. A more reliable earlier NEC diagnosis is critical for earlier intervention to arrest the disease (1). Hoffsten et al. investigated 189 early postnatal comprehensive biomarkers utilizing a proximity extension assay, including inflammatory, vascular proteomic, and oncological parameters. Altered gut microbiota is a risk factor for the development of NEC, and it is especially pronounced in premature NEC infants. Here, we infer that comprehensive analysis of the alterations in the intestinal microbial population might provide valuable diagnostic information to detect this disease early. However, due to the sophistication of bioinformatics analysis and different platforms in terms of data acquisition, variable results have been presented for intestinal microbiota analysis from study to study (3). Which select bacteria within the fecal sample are more appropriate for NEC detection should be further investigated.

Although the present evidence to guide microbiome management strategies remains controversial, the current collection may provide additional further understanding for optimization of the components of the microbiome in this field in preterm infants.

## Author Contributions

CG and YS designed and developed this Research Topic, contributed to the accompanying editorial, contributed to the current editorial, and approved the current submission in the present form. Both authors conferred great efforts to evaluate the published manuscripts.

## Conflict of Interest

The authors declare that the research was conducted in the absence of any commercial or financial relationships that could be construed as a potential conflict of interest.

## Publisher's Note

All claims expressed in this article are solely those of the authors and do not necessarily represent those of their affiliated organizations, or those of the publisher, the editors and the reviewers. Any product that may be evaluated in this article, or claim that may be made by its manufacturer, is not guaranteed or endorsed by the publisher.
